# Supplementary material for: Survival mechanism of pancreatic tumor bacteria and their ability to metabolize chemotherapy drugs
Source: Microbiol Spectr. 2025 Aug 12;13(9):e01820-25. doi: 10.1128/spectrum.01820-25 (PMC12403566; doi:10.1128/spectrum.01820-25)
Supplement: Supplemental tables and figures — Tables S1 to S3, and Fig. S1 to S5. [file spectrum.01820-25-s0001.docx]

**SI Table 1. Growth curve assessment for IPMN derived bacterial strains.**

| **Isolation** | **Bacterial strains** | **Doubling time (mins)** | **Lag phase (mins)** | **Log phase (mins)** | **Stationary phase (mins)** |
| --- | --- | --- | --- | --- | --- |
| IC | K. pneumoniae (C2) | 38 | 0-75 | 75-180 | 180-1200 |
| IC | S. anginosus (C2) | 30 | 0-105 | 105-195 | 195-1200 |
| IC | E. cloacae (C2) | 48 | 0-75 | 75-195 | 195-1200 |
| IC | E. faecalis (C1) | 37 | 0-90 | 90-195 | 195-1200 |
| IC | S. maltophili (C1) | 36 | 0-75 | 75-180 | 180-1200 |
| HGD | K. oxytoca (H1) | 34 | 0-75 | 75-180 | 180-1200 |
| HGD | C. freundii (H1) | 40 | 0-90 | 90-195 | 195-1200 |
| HGD | E. cloacae (H2) | 51 | 0-75 | 75-210 | 210-1200 |
| HGD | E. faecium (H2) | 33 | 0-90 | 90-210 | 210-1200 |
| HGD | S. anginosus (H2) | 34 | 0-75 | 75-210 | 210-1200 |
| LDG | K. aerogenes (L1) | 37 | 0-75 | 75-180 | 180-1200 |
| LDG | E. faecalis (L2) | 36 | 0-105 | 105-195 | 195-1200 |
| LDG | E. cloacae (L2) | 61 | 0-165 | 165-345 | 345-1200 |
| Control | E. coli d12 | 36 | 0-105 | 105-240 | 240-1200 |
| Control | E. coli 25922 | 30 | 0-150 | 150-270 | 270-1200 |


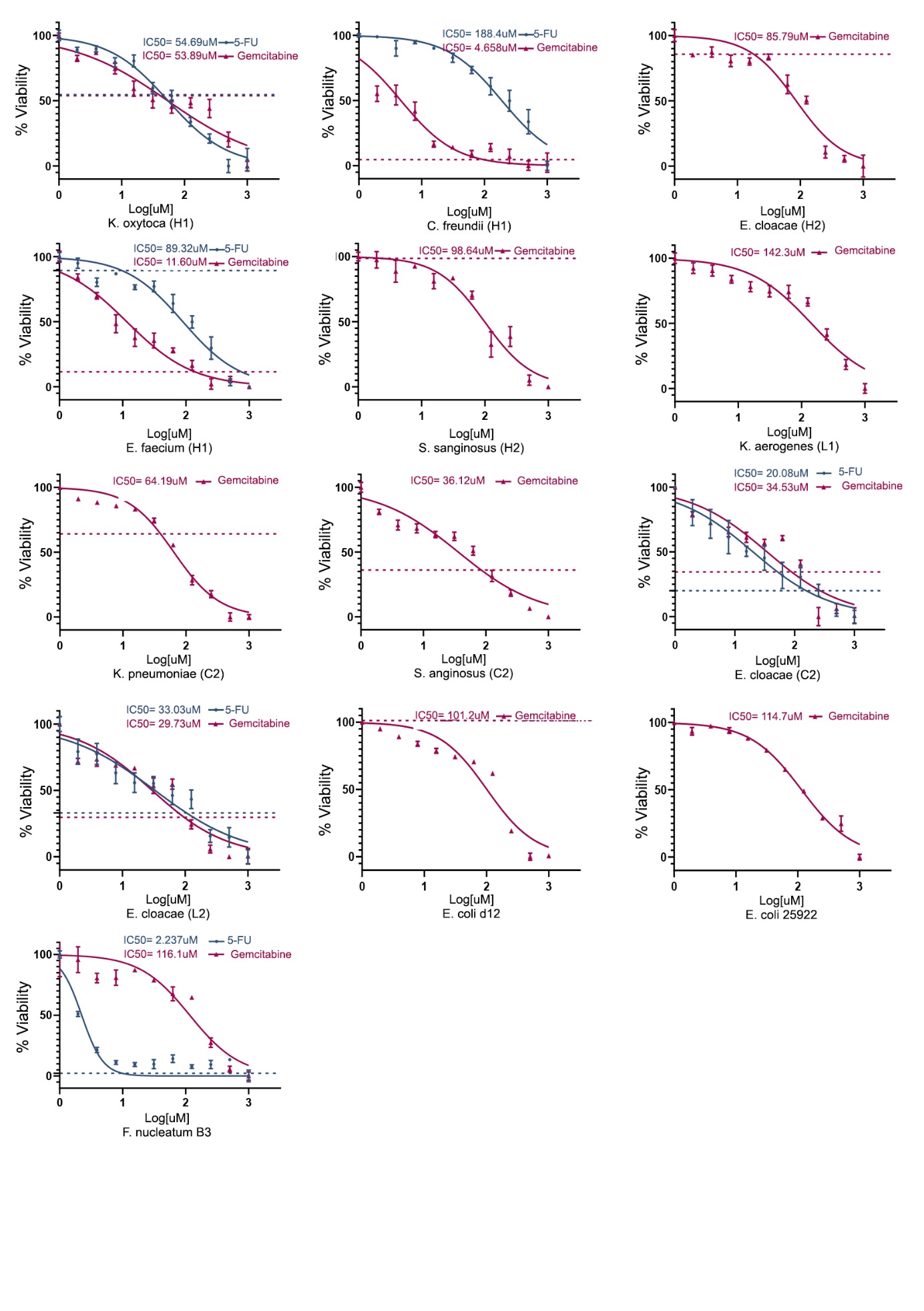


**SI-1. IC50 for chemotherapeutics drugs 5-FU and gemcitabine on bacteria strains.**

**SI. Table 2 IC50 for IPMN derived bacterial strains.**

| **Isolation** | **Bacterial Strains** | **Class** | **Gemcitabine (**µ**M)** | | **5-FU (**µ**M)** |
| --- | --- | --- | --- | --- | --- |
| IC | K. pneumoniae (C2) | Gammaproteobacteria | 64.19 | Resistant | |
| IC | S. anginosus (C2) | Bacilli | 36.12 | Resistant | |
| IC | E. cloacae (C2) | Gammaproteobacteria | 34.53 | 20.08 | |
| IC | E. faecalis (C1) | Bacilli | 68.86 | 24.37 | |
| IC | S. maltophili (C1) | Gammaproteobacteria | 89.98 | Resistant | |
| HGD | E. cloacae (H2) | Gammaproteobacteria | 85.79 | Resistant | |
| HGD | E. faecium (H2) | Bacilli | 11.6 | 89.32 | |
| HGD | S. anginosus (H2) | Bacilli | 98.64 | Resistant | |
| HGD | K. oxytoca (H1) | Gammaproteobacteria | 53.89 | 54.69 | |
| HGD | C. freundii (H1) | Gammaproteobacteria | 4.658 | 188.4 | |
| LDG | K. aerogenes (L1) | Gammaproteobacteria | 142.3 | Resistant | |
| LDG | E. faecalis (L2) | Bacilli | 18.15 | Resistant | |
| LDG | E. cloacae (L2) | Gammaproteobacteria | 29.73 | 33.03 | |
| Control | E. coli d12 | Gammaproteobacteria | 101.2 | Resistant | |
| Control | E. coli 25922 | Gammaproteobacteria | 114.7 | Resistant | |
| Control | F. nucleatum O1 | Fusobacteriaceae | Resistant | Resistant | |
| Control | F. nucleatum B2 | Fusobacteriaceae | Resistant | Resistant | |
| Control | F. nucleatum B3 | Fusobacteriaceae | 116.1 | 2.337 | |


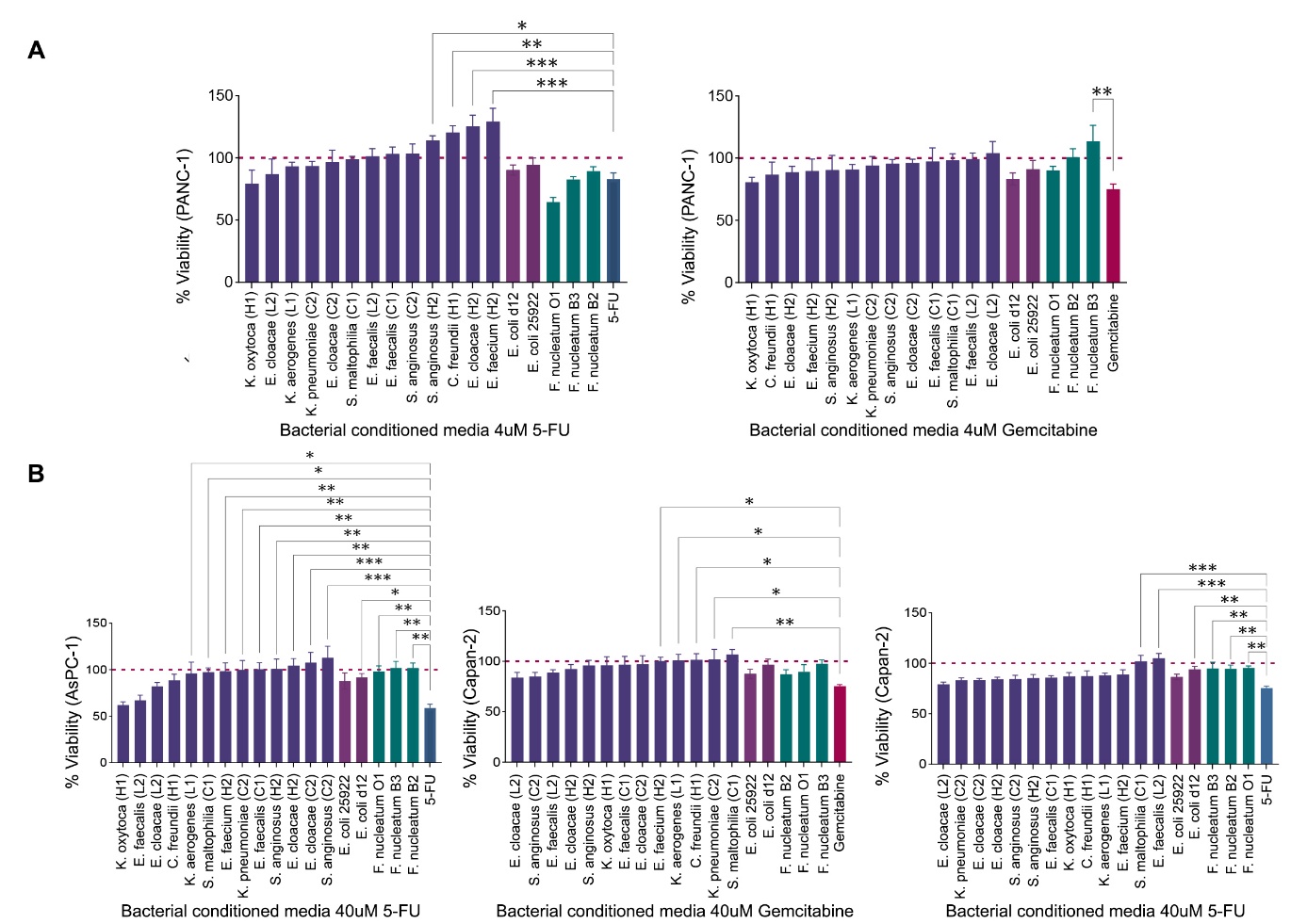


**SI-2. Drug degradation of 5-FU and gemcitabine (4uM and 40uM) into nontoxic products on pancreatic cancer cells (Y-axis) via indicated bacterial strains (X-axis).**

**SI Table 3. WGS characterization of IPMN derived microbes and 5-FU-R strains.**

| **Characteristics** | **E. cloacae (H2)** | **E. cloacae (C2)** | **E. cloacae (L2)** | | **K. oxytoca (H1)** | **E. faecalis (L2)** | **S. anginosus (H2)** | **S. anginosus (C2)** | **E. Faecium (H2)** | **E. coli (MG1655)** | **B. ovatis** |
| --- | --- | --- | --- | --- | --- | --- | --- | --- | --- | --- | --- |
| **Genome Statistics** | | |  |  | |  |  |  |  |  |  |
| **Contigs** | 25 | 49 | 58 | 64 | | 37 | 41 | 55 | 114 | 106 | 87 |
| **Genome Length** | 4715723 | 4902509 | 4612969 | 5785559 | | 2838465 | 1859888 | 1819384 | 2641801 | 4550114 | 6363341 |
| **GC Content** | 56.02929 | 55.489933 | 56.195694 | 55.048615 | | 37.45239 | 38.795563 | 38.91883 | 38.153973 | 50.745388 | 41.859238 |
| **Contig L50** | 3 | 5 | 5 | 7 | | 4 | 6 | 5 | 18 | 13 | 10 |
| **Contig N50** | 581429 | 396778 | 283807 | 302791 | | 185180 | 119335 | 97614 | 51970 | 124426 | 217210 |
| **Annotation Statistics** | | |  |  | |  |  |  |  |  |  |
| **tRNA** | 67 | 67 | 67 | 72 | | 49 | 29 | 34 | 48 | 73 | 63 |
| **rRNA** | 4 | 5 | 6 | 4 | | 3 | 3 | 3 | 3 | 2 | 2 |
| **CDS** | 4469 | 4779 | 4354 | 5549 | | 2725 | 1853 | 1861 | 2739 | 4474 | 5018 |
| **CDS Ratio** | 0.9476808 | 0.97480696 | 0.94386065 | 0.95911217 | | 0.9600259 | 0.9962965 | 1.0228736 | 1.0367928 | 0.9832721 | 0,78857946 |
| **Hypothetical CDS** | 463 | 611 | 421 | 731 | | 548 | 363 | 330 | 753 | 259 | 1881 |
| **Hypothetical CDS Ratio** | 0.23718953 | 0.2594685 | 0.23587506 | 0.2636511 | | 0.31412843 | 0.28278467 | 0.26706073 | 0.3592552 | 0.25033528 | 0.4693105 |
| **PLFAM CDS** | 4323 | 4620 | 4279 | 5386 | | 2681 | 1778 | 1798 | 2525 | 4403 | 4817 |
| **PLFAM CDS Ratio** | 0.9673305 | 0.96672946 | 0.98277444 | 0.97062534 | | 0.9838532 | 0.9595251 | 0.96614724 | 0.9218693 | 0.98413056 | 0.9599442 |
| **Genome Quality** | | |  |  | |  |  |  |  |  |  |
| **Coarse Consistency** | 99.2 | 99.4 | 98.9 | 99 | | 99.6 | 99.5 | 99.4 | 98.5 | 99.9 | 99.5 |
| **Fine Consistency** | 98.5 | 97.9 | 98.1 | 96.8 | | 99 | 99 | 98.9 | 97.6 | 99.7 | 98.9 |


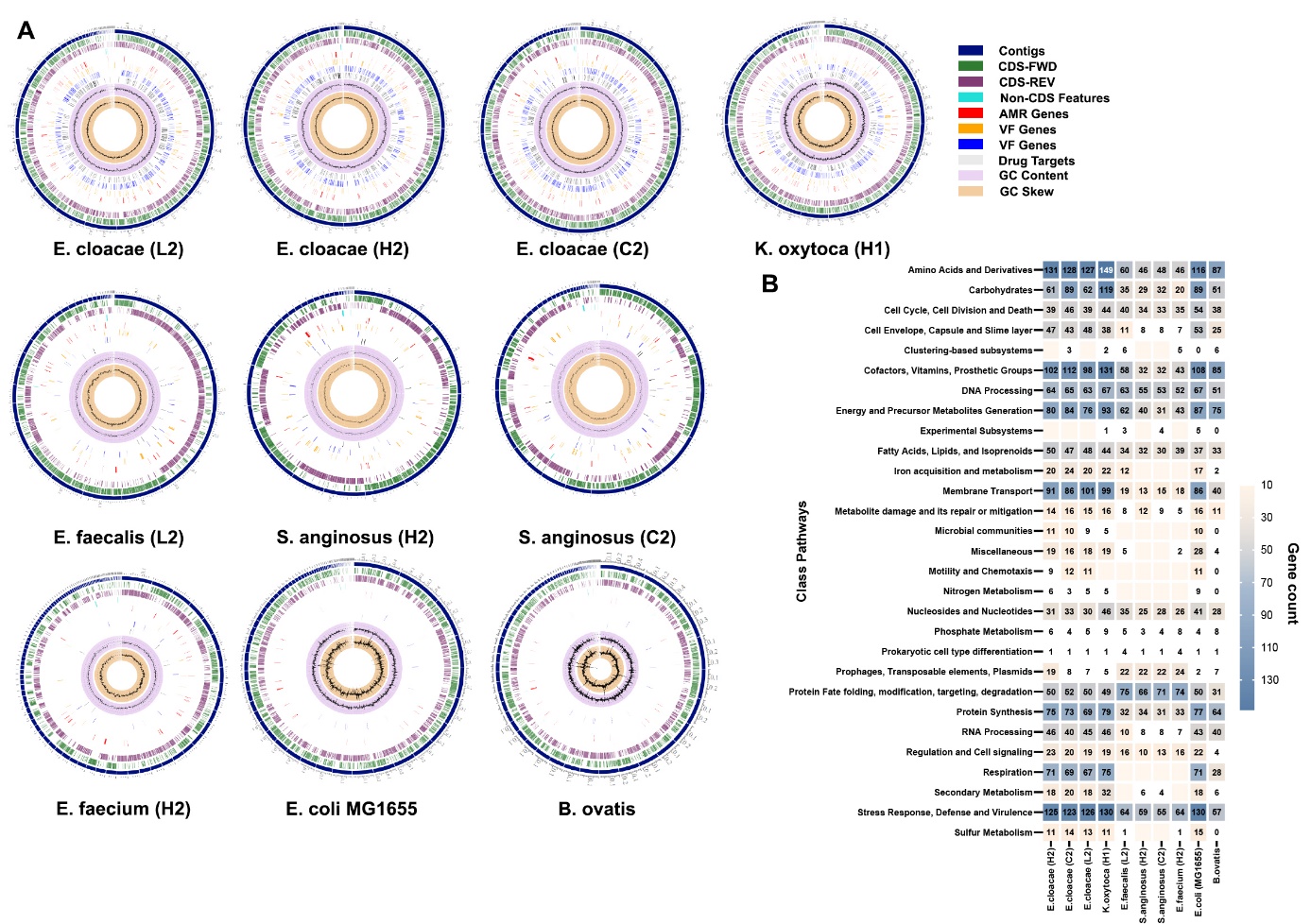


**SI-3. Circular diagram of WGS for IPMN derived strains.**


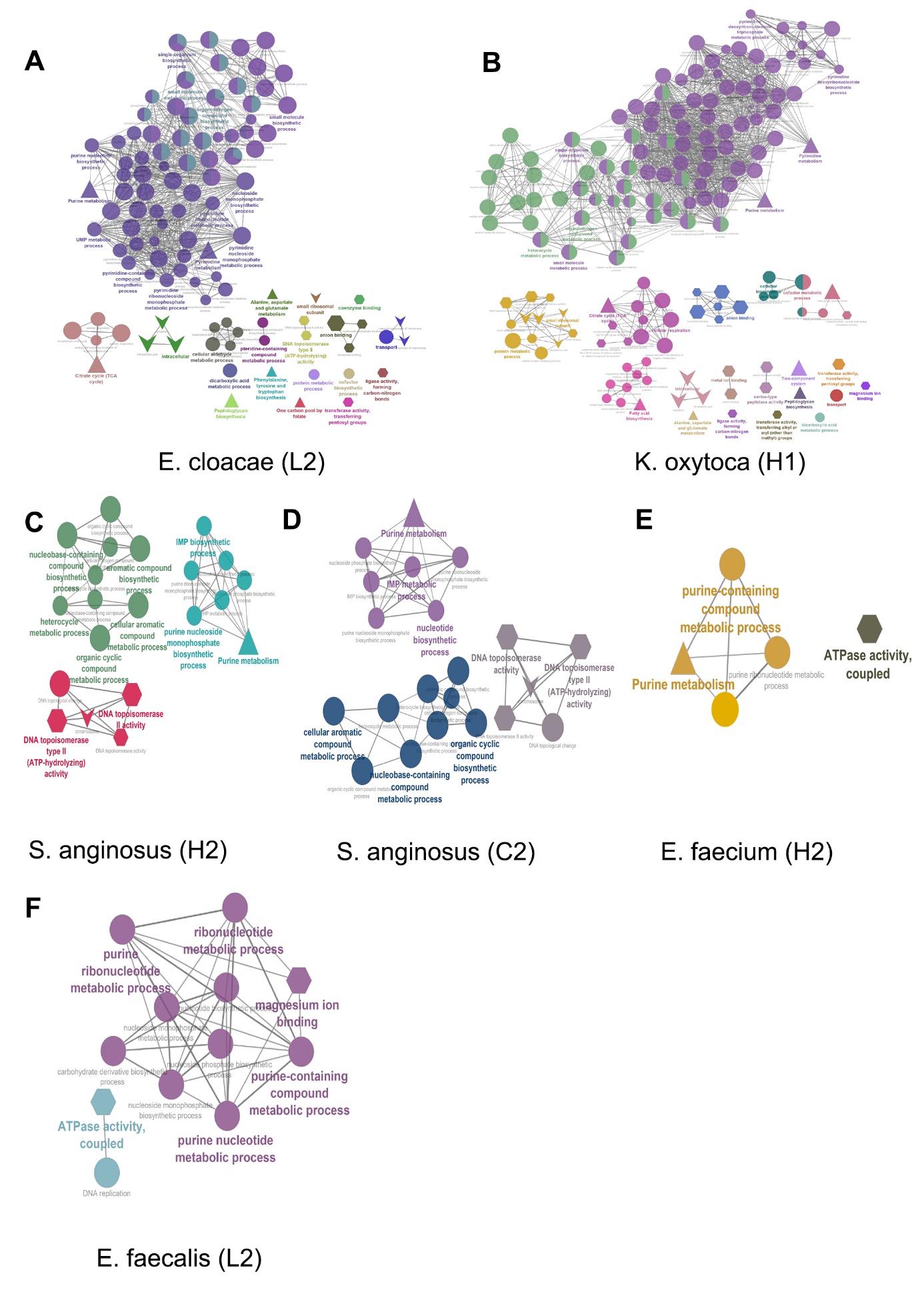


**SI 4. Pathway enrichment for specialty genes.**


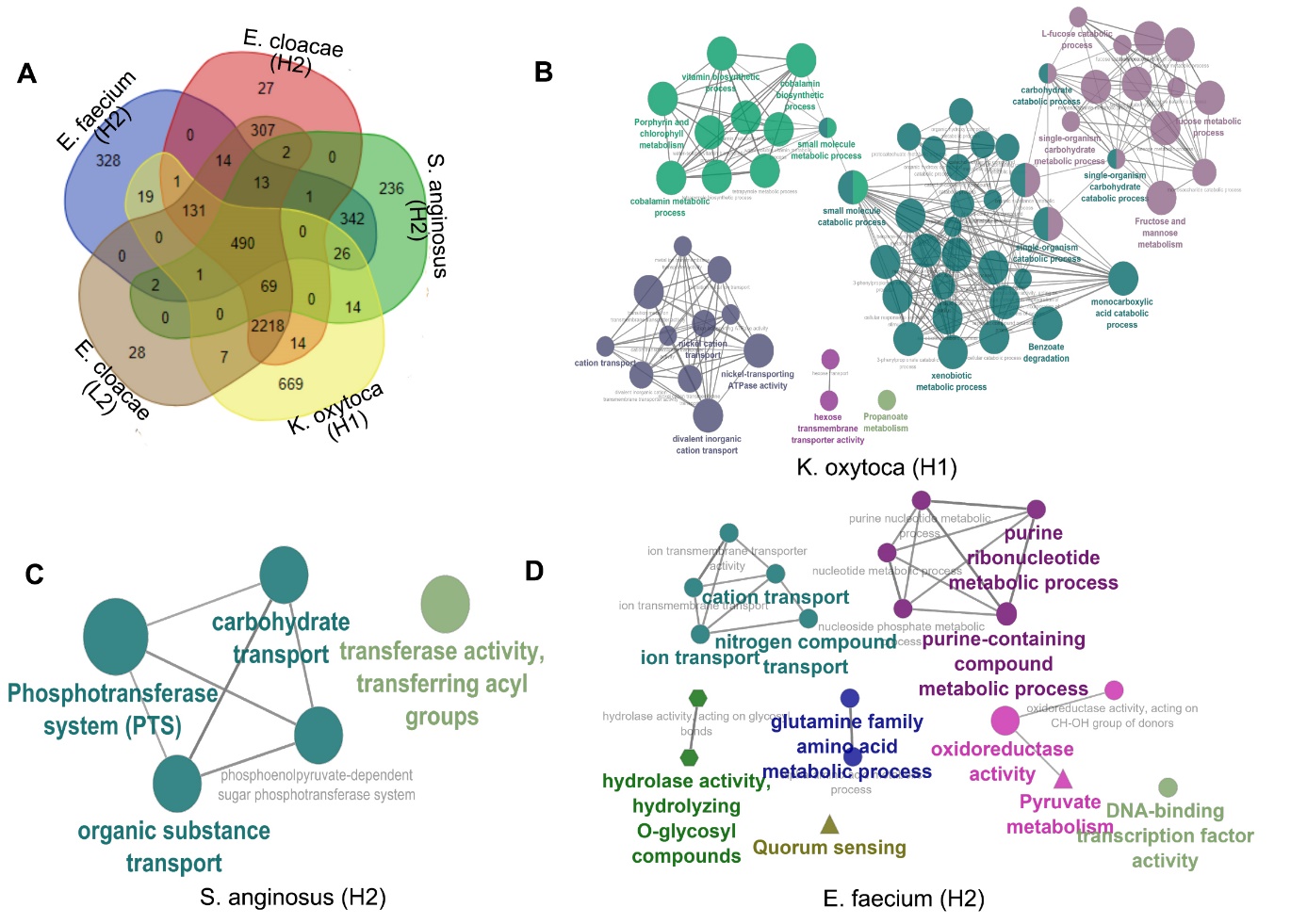


**SI 5. Genomic comparison between E. cloacae (L2) and HGD strains.**
