# Supplementary figures and images for: Survival mechanism of pancreatic tumor bacteria and their ability to metabolize chemotherapy drugs
Source: Microbiol Spectr. 2025 Aug 12;13(9):e01820-25. doi: 10.1128/spectrum.01820-25 (PMC12403566; doi:10.1128/spectrum.01820-25)

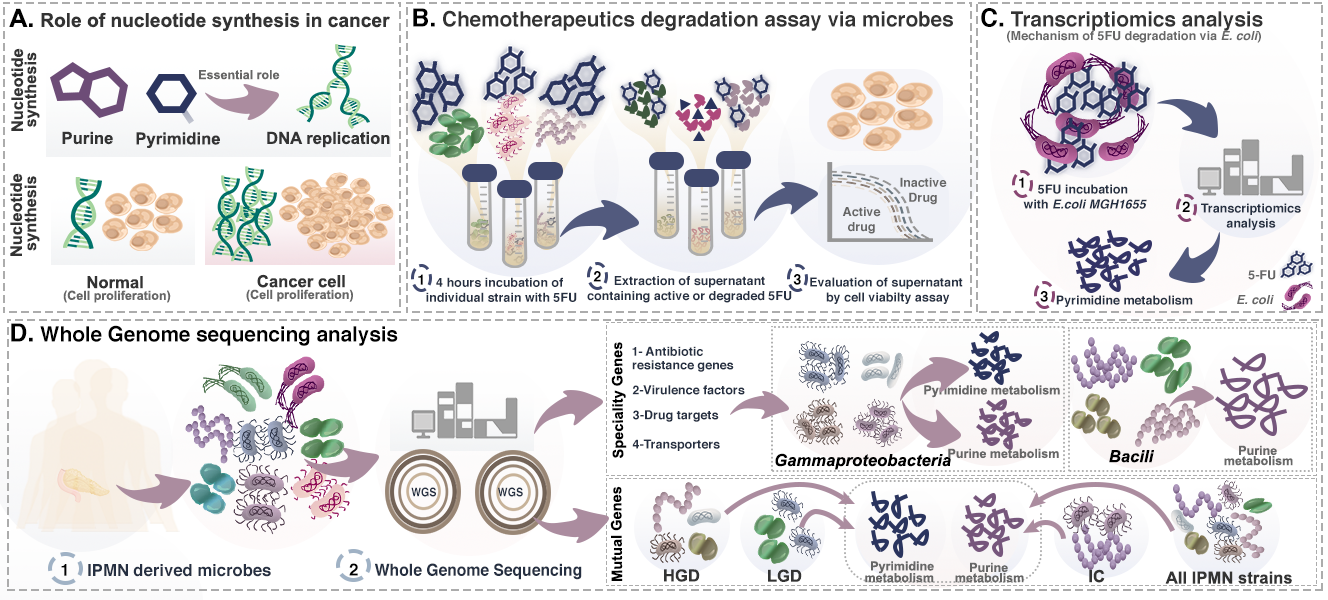

Supplement: Graphical abstract [file spectrum.01820-25-s0002.tif]
